# Epidemiological and genetic characterization of *Clostridium butyricum* cultured from neonatal cases of necrotizing enterocolitis in China

**DOI:** 10.1017/ice.2019.289

**Published:** 2020-08

**Authors:** Yinping Dong, Ying Li, Di Zhang, Scott Nguyen, Nikunj Maheshwari, Yujie Hu, Yu Cao, Fengqin Li, Séamus Fanning

**Affiliations:** 1 NHC Key Laboratory of Food Safety Risk Assessment, China National Center for Food Safety Risk Assessment, Beijing, PR China; 2 Children’s Hospital Affiliated with Capital Institute of Pediatrics, Beijing, PR China; 3 School of Public Health, Physiotherapy & Sports Science, University College of Dublin, Dublin, Ireland

## Abstract

**Objective:**

Laboratory-based characterization and traceback of *Clostridium butyricum* isolates linked to outbreak cases of neonatal necrotizing enterocolitis (NEC) in a hospital in China.

**Methods:**

In total, 37 samples were collected during the NEC outbreak. Classical bacteriological methods were applied to isolate and identify *Clostridium* spp. Meanwhile, 24 samples collected after an outbreak were similarly tested. All *Clostridium* isolates were identified to species level as either *C. butyricum* or *C. sporogenes*. These isolates were subsequently subtyped using pulsed-field gel electrophoresis (PFGE). Genomic DNA was purified from 2 representative *C. butyricum* isolates and sequenced to completion.

**Results:**

Of 37 samples collected during the NEC outbreak, 17 (45.95%) were positive for *Clostridium* spp. One species, *C. butyricum*, was cultured from 10 samples. Another species cultured from 2 other samples was identified as *C. sporogenes*. Both of these species were cocultured from 5 samples. Pulsotyping showed that the 15 *C. butyricum* and the 7 *C. sporogenes* isolates produced indistinguishable DNA profiles. No NEC cases were reported after disinfection following the outbreak, and all samples collected after the outbreak were negative for *Clostridium* spp. Whole-genome sequencing (WGS) indicated that sialidase, hemolysin, and enterotoxin virulence factors were located on the chromosomes of 2 *C. butyricum* isolates.

**Conclusions:**

The outbreak of NEC was epidemiologically linked to *C. butyricum* contamination within the hospital. This is the first report of an NEC outbreak associated with *C. butyricum* infection in China.

Necrotizing enterocolitis (NEC) is a severe and life-threatening, acquired gastrointestinal disorder among preterm neonates, especially infants of very low birth weight (< 1,500 g).^1^^–^^3^ NEC is characterized by abdominal distension, blood in the stool, portal venous gas, pneumatosis intestinalis, and pneumoperitoneum, with reported mortality ranging from 20% to 50%.^1^ Although NEC pathophysiology remains unclear, prematurity, enteral feeding strategies, gut bacterial colonization, and inappropriate proinflammatory response are major factors implicated in NEC development.^4^ Several causative microorganisms including viruses, gram-negative bacilli, and *Clostridium* spp have been proposed.^5^ No etiologic agent has been definitively established; nonetheless, the most commonly implicated bacteria include members of the *Clostridium* genus, especially *C. butyricum*.^6^ Cytotoxic *C. butyricum* isolates obtained from a larger population of preterm neonates with NEC strengthen the etiology of *C. butyricum* in cases of NEC, independent of intrinsic factors.^7^^,^^8^ NEC-like lesions produced by *C. butyricum* in animal models as well as in Caco-2 cell lines have also been successfully duplicated, further demonstrating the cytotoxic activity of this bacterium.^9^^–^^12^ Genome sequencing of the isolates cultured from specimens linked to NEC patients allowed the identification of genes encoding polypeptides that are highly similar to hemolysins shared by *Brachyspira hyodysenteriae*.^13^

In this study, we investigated an outbreak of NEC in Shandong Province, China. We carried out the laboratory-based epidemiological traceability and genetic characterization of *C. butyricum* isolated from specimens linked to NEC outbreak to explore the potential role of *C. butyricum* in the pathogenesis of NEC.

## Materials and methods

### Ethics statement

Experiments related to infant fecal specimens were performed in accordance with the written informed consent from each patient’s guardian. The study protocol was approved by the Ethics Committee of China National Center for Food Safety Risk Assessment.

### Case overview

From October through November 2016, an outbreak of NEC occurred in a maternal and child health-care hospital in Shandong Province, China. Infants who presented with bloody stools, abdominal distension with or without drowsiness, apnea, hypotonia, and/or pneumatosis intestinalis were diagnosed as NEC cases. In total, 15 cases were recorded within 1 month in the neonatal intensive care unit (NICU), and all patients were neonates. Among them, 5 were premature neonates born in this hospital and were subsequently diagnosed with NEC 4–6 days after birth. The other 10 NEC cases were neonates delivered at full term in other hospitals who had been admitted to the onset hospital (where the NEC index was reported) as a result of unrelated complications. All 10 of these neonates were discharged from the onset hospital having made a full recovery from their primary disease, after which they were all subsequently readmitted to the onset hospital. All 15 NEC cases had the same clinical symptoms and signs, including bloody stools and abdominal distension without fever and infectious sepsis. These infants were fed with either breast milk or infant formula powder prior to their NEC diagnosis.

### Sample collection

In total, 61 samples, including 37 taken from sources linked to the NEC outbreak and 24 others (including 1 breast milk and 23 environmental samples) after disinfectant interventions following the outbreak, respectively, were collected for microbiological analysis. Among these 15 NEC patients, 11 had been discharged originally from the hospital at the time of sample collection. Another 2 patients who remained hospitalized at the index hospital did not produce sufficient stool samples for testing due to receiving fasting and water-free treatment strategies after being diagnosed as NEC. Only 2 stool samples were successfully collected from 2 NEC case patients (cases A and B). In addition, 8 hand swabs were obtained from 8 nurses responsible for taking care of these children in the ward at the time of sampling. All of these samples were collected by swabbing a 10-cm × 10-cm surface area of these objects with surface samplers (UXL100, 3M, Cottage Grove, MN) before depositing them into tubes containing buffered peptone water (BPW). Further details of the sampling methods are included in Table 1.

**Table 1. tbl1:** The Information of Samples Analyzed

Sampling Time	Sample Source	Sample Name	No. of Samples
During an NEC outbreak	Related to 2 NEC cases	Stools	2
		Leftover powdered infant formula	2
		Breast milk	2
	Related to a non-NEC case	Breast milk	1
	Ward environment	Swabs of inner wall of non-NEC infant incubator	4
		Swab of inner wall of the breast milk bag	1
		Swab of inner wall of the NEC-infant incubator	1
		Swab of handle of the NEC-infant incubator	1
		Swab of inner wall of refrigerator	1
		Swab of milk-preparation desk	1
		Swab of treatment table	1
		Swab of ECG monitor	1
		Swab of infusion pump	1
		Swab of stethoscope	1
		Swab of public phone	1
		Swab of baffle of radiation probe	1
	Medical staff	Hand swabs	8
	Articles for neonate daily use	Swab of inner wall of a box for holding breast milk bag	1
		Swabs of inside of the feeding bottles	2
		Swabs of nipple of feeding bottles	2
		Swab of outside of the cap of a feeding bottle	1
		Water	1
	Total		37
After interventions following outbreak	Related to the non-NEC case	Breast milk	1
	Ward environment	Swabs of inner wall of the case patient’s incubator	2
		Swab of inner wall of refrigerator	1
		Swab of ECG monitor	1
		Swab of infusion pump	1
		Swab of baffle of radiation probe	1
		Sink	1
		Swab of inner wall of tourniquet box	1
		Swabs of public phone	2
		Swabs of computer mouse	2
		Swabs of computer keyboard	2
	Medical staff	Hand swabs	2
	Articles for neonate daily use	Swab of inner wall of feeding bottle cabinet	1
		Swab of inner wall of a box for holding breast milk bag	1
		Swab of inside of the feeding bottle	1
		Swab of nipple of a feeding bottle	1
		Water	1
		Powdered infant formula	2
	Total		24
Total			61

### Laboratory analysis

*Isolation, identification of* *Clostridium* spp. *Clostridium* was isolated in accordance with the US Food and Drug Administration’s *Bacteriological Analytical Manual* (Chapter 17: *Clostridium botulinum*).^14^ Presumptive colonies were selected for gram staining, microscopic examination, API 20A and VITEK2 (bioMérieux, Marcy l’Etoile, France) biochemical testing, and 16S rRNA gene sequencing in accordance with the recommended procedures.

*PFGE molecular subtyping*. Presumptive *Clostridium* spp cultured from the samples described above were purified, and PFGE subtyping was performed according to the previously published method.^15^

*Whole-genomic sequencing*. Two *C. butyricum* isolates cultured from samples of the patient’s stool and a swab taken from the inner wall of NEC-infant’s incubator coded F1-b and F5-b, respectively, were recovered, followed by genomic DNA purification. Whole-genome sequencing was performed using the Pacific Biosciences RS II platform (SMRT, Pacific Biosciences, Menlo Park, CA). *De novo* assembly of the reads was carried out using Hierarchical Genome Assembly Process (HGAP) version 3.0 software module.

### Bioinformatical analysis

The 2 complete *C. butyricum* genomes sequenced in this study were compared with a reference *C. butyricum* KNU-L09 genome (accession no. NZ_CP013252). A bioinformatics framework (seq-seq-pan), designed to construct a pangenome from a set of aligned genomes, was used to construct a *C. butyricum* pangenome from all 3 genomes.^16^ A BLAST ring image generator (BRIG) was used to construct a comparative genomic representation of the 3 *C. butyricum* genomes against the pangenome.^17^ Putative prophages were identified using the PHASTER web server.^18^ Genomic islands and other mobile genetic elements (MGE) were manually inspected and determined by Mauve alignment of the genomes in Geneious version 8.1.6 software (Biomatters, Auckland, New Zealand).^19^ The virulence factor database (VFDB, http://www.mgc.ac.cn/VFs/main.htm) was used to predict the presence of virulence factors in the genomes of the sequenced strains. *Clostridium butryicum*sequences were downloaded from the National Center for Biotechnology Information (NCBI) using the ncbi-genome-download script (https://github.com/kblin/ncbi-genome-download). Parsnp from the Harvest suite was used to construct a phylogenetic tree of the *C. butyricum* sequences in conjunction with the 2 sequenced strains in this study.^20^ Parsnp was run with default options, and *C. butryicum* KNU-L09 was included as the reference for the phylogenetic tree; Evolview 2 was used to visualize the tree.^21^ Nucleotide sequences and statistics regarding 2 *C. butyricum* isolates were submitted to GenBank.

## Results

### *Clostridium* isolation and identification

In total, 37 samples were collected during the NEC outbreak, and 17 (45.95%) were positive for a presumptive *Clostridium*. Also, 15 samples (40.50%) were contaminated with *C. butyricum*, as determined by gram staining, microscopic examination, biochemical testing, and 16S rRNA gene sequencing. These positive samples included the leftover powdered infant formula (PIF) sample initially consumed by the neonates linked to the NEC cases, feces of NEC patients, swab samples taken from the hands of the attending medical staff, disposable items for neonate daily use, breast milk bags, medical equipment used in the ward (eg, infusion pump, an electrocardiogram (ECG) monitor, stethoscope, telephone, refrigerator, as well as handle and inner wall surfaces of the neonate incubator) (Table 2). Of 37 samples, 5 (13.5%) including the feces of 2 NEC patients, swab samples taken from the inner wall of a refrigerator, the door handle of the incubator as well as disposable items for daily use were cocontaminated with both *C. butyricum* and *C. sporogenes*. Two samples (5.4%) were positive for *C. sporogenes* alone (Table 2).

**Table 2.** The Results of *Clostridium* spp Isolation and Identification

Sample Source	Sample Code	Sample Name	Identification Results			
			Biochemical Test	16S rRNA Gene Sequencing	Final	Strain Code
Samples associated with case A	F1	Stool	*C. butyricum*	*C. butyricum*	*C. butyricum*	F1-b
			*C. sporogens*	*C. sporogens*/ *C. botulinum*	*C. sporogens*	F1-s
	F4	Swab of inner wall of breast milk bag	*C. sporogens*	*C. sporogens*/ *C. botulinum*	*C. sporogens*	F4-s
	F5	Swab of inner wall of NEC-infant incubator	*C. butyricum*	*C. butyricum*	*C. butyricum*	F5-b
	F6	Swab of handle of NEC-infant incubator	*C. butyricum*	*C. butyricum*	*C. butyricum*	F6-b
			*C. sporogens*	*C. sporogens*/ *C. botulinum*	*C. sporogens*	F6-s
Samples associated with case B	F7	Stool	*C. butyricum*	*C. butyricum*	*C. butyricum*	F7-b
			*C. sporogens*	*C. sporogens*/ *C. botulinum*	*C. sporogens*	F7-s
	F8	Powdered infant formula	*C. butyricum*	*C. butyricum*	*C. butyricum*	F8-b
Samples associated with non-NEC infant	F36	Breast milk	*C. sporogens*	*C. sporogens*/ *C.botulinum*	*C. sporogens*	F36-s
Swabs of the medical equipment in the ward	F13	Inner wall of refrigerator	*C. butyricum*	*C. butyricum*	*C. butyricum*	F13-b
			*C. sporogens*	*C. sporogens*/ *C. botulinum*	*C. sporogens*	F13-s
	F16	ECG monitor	*C. butyricum*	*C. butyricum*	*C. butyricum*	F16-b
	F17	Infusion pump	*C. butyricum*	*C. butyricum*	*C. butyricum*	F17-b
	F18	Stethoscope	*C. butyricum*	*C. butyricum*	*C. butyricum*	F18-b
	F19	Public phone	*C. butyricum*	*C. butyricum*	*C. butyricum*	F19-b
Hand swabs of medical staffs	F21	Staff 1	*C. butyricum*	*C. butyricum*	*C. butyricum*	F21-b
	F22	Staff 2	*C. butyricum*	*C. butyricum*	*C. butyricum*	F22-b
	F23	Staff 3	*C. butyricum*	*C. butyricum*	*C. butyricum*	F23-b
	F27	Staff 7	*C. butyricum*	*C. butyricum*	*C. butyricum*	F27-b
Swabs of articles for neonate daily use	F29	Inner wall of a box for holding breast milk bag	*C. butyricum*	*C. butyricum*	*C. butyricum*	F29-b
			*C. sporogens*	*C. sporogens*/ *C. botulinum*	*C. sporogens*	F29-s

**Fig. 1.**
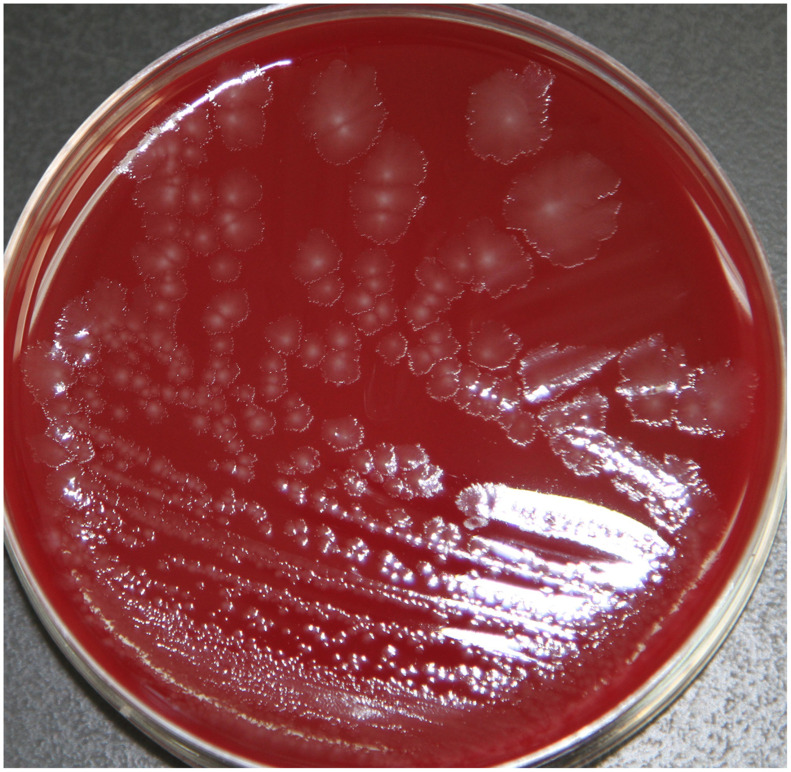
Morphology on Columbia blood agar of *C. butyricum*.

*Clostridium butyricum* cultured from NEC-related samples produced colonies were white in color, semitransparent, and had irregular contours and rhizoid colonies on Columbia blood agar (Fig. 1). When subjected to a gram staining observed under high-phase microscopy, these bacterial cells resembled a tennis-racket shape in which the oval-shaped spores were greater in width than the bacterium.

Normally, in the NICU in this hospital, only the floor and desk surfaces are disinfected once daily with sodium trichloroisocyanurate at a concentration of 500 mg/L. After this outbreak, based on our laboratory results, the hospital implemented control measures including thorough daily disinfection of the wards, hands of the medical staff, all articles for neonate daily use, and infant feeding supplies. In total, 24 swab samples were subsequently taken from the ward environment, the hands of the attending medical staff, and disposable items for neonate daily use after interventions (Table 1). All were negative for *Clostridium*[Table tbl1]. No NEC cases were recorded after disinfection. Therefore, the outbreak of NEC was suspected to be correlated with the contamination of *Clostridium*, especially *C. butyricum*.

### PFGE-based pulsotyping

**Fig. 2.**
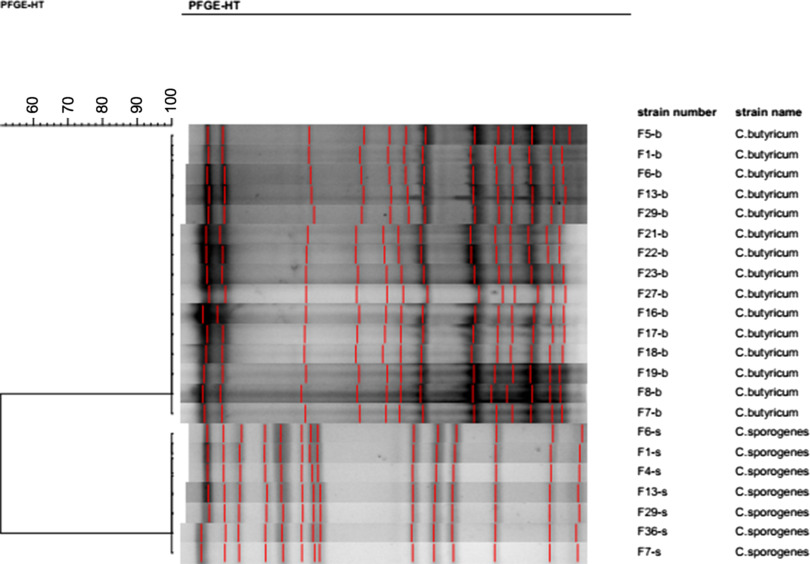
Pulsotypes of 15 *C. butyricum* and 7 *C. sporogenes*isolates cultured from samples with SmaI restrictive endonuclease.

All 15 isolates of *C. butyricum* cultured in this investigation were subjected to pulsotyping, and a single profile was recorded (Fig. 2). This finding suggests that all *C. butyricum* isolates shared the same pulsotype, and, furthermore, that both PIF intended for infant consumption and the ward environment were contaminated with the same strain of *C. butyricum*. According to the PFGE analysis, the 7 *C. sporogenes* isolated from this investigation shared the same profile (Fig. 2). Thus, the *C. sporogenes* isolated from the infant’s intestine may have been acquired from the contaminated breast milk.

### Whole-genome sequencing of two representative isolates of *C. butyricum*

**Fig. 3.**
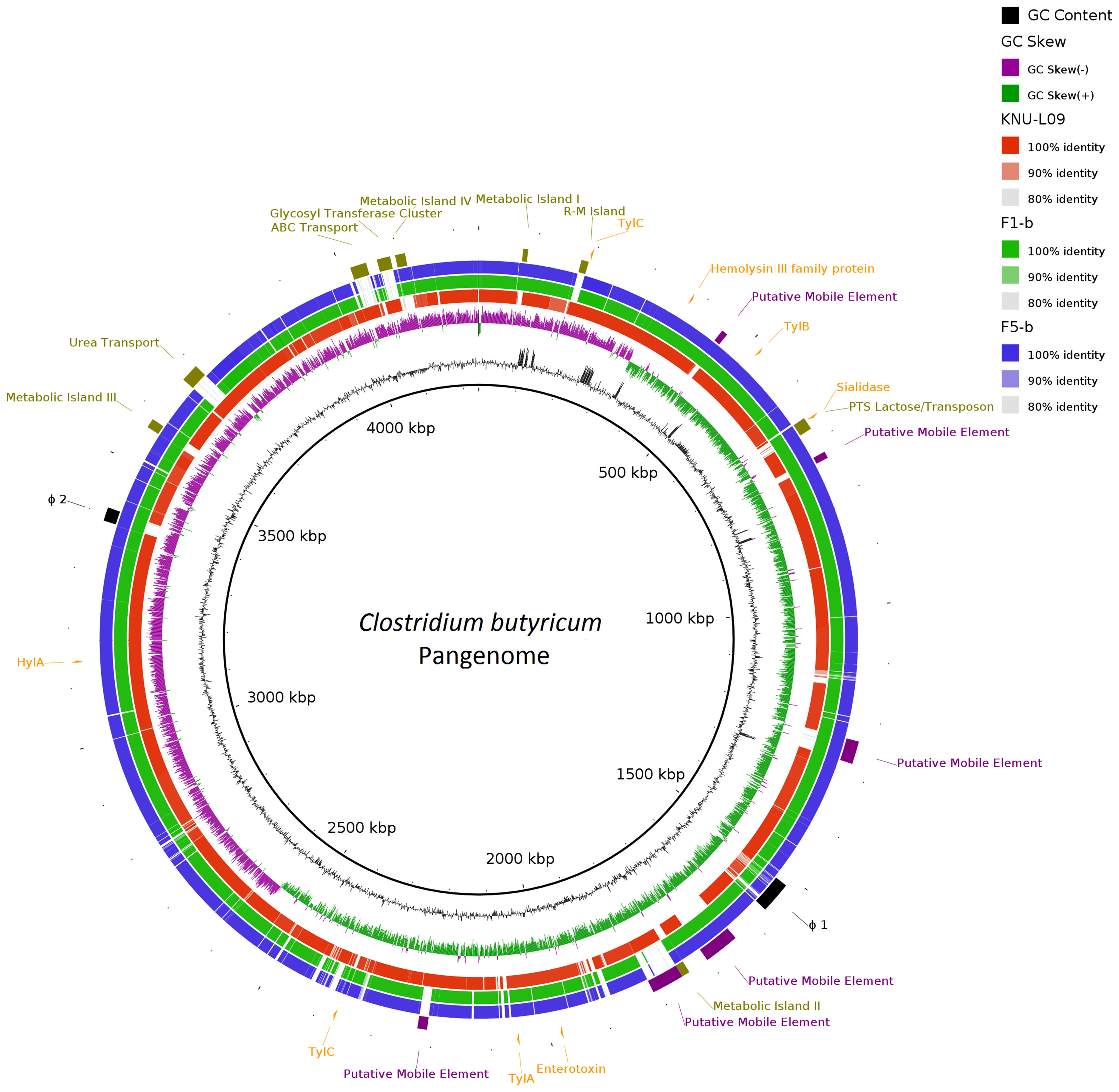
Major virulence genes of *C. butyricum* F1-b and F1-5 compared with reference strain *C. butyricum* KNU-L09 (accession no. NZ_CP013252). Beginning from the inside out represents the following features: GC content (black); GC skew (green and purple); *C. butyricum* KNU-L09 genome (red); *C. butyricum* F1-b genome (green); *C. butyricum* F5-b genome (blue); transport genes (shown in green font), putative mobile element (shown in purple font), phage genes (shown in black font) and virulence factor genes (shown in orange font).

**Fig. 4.**
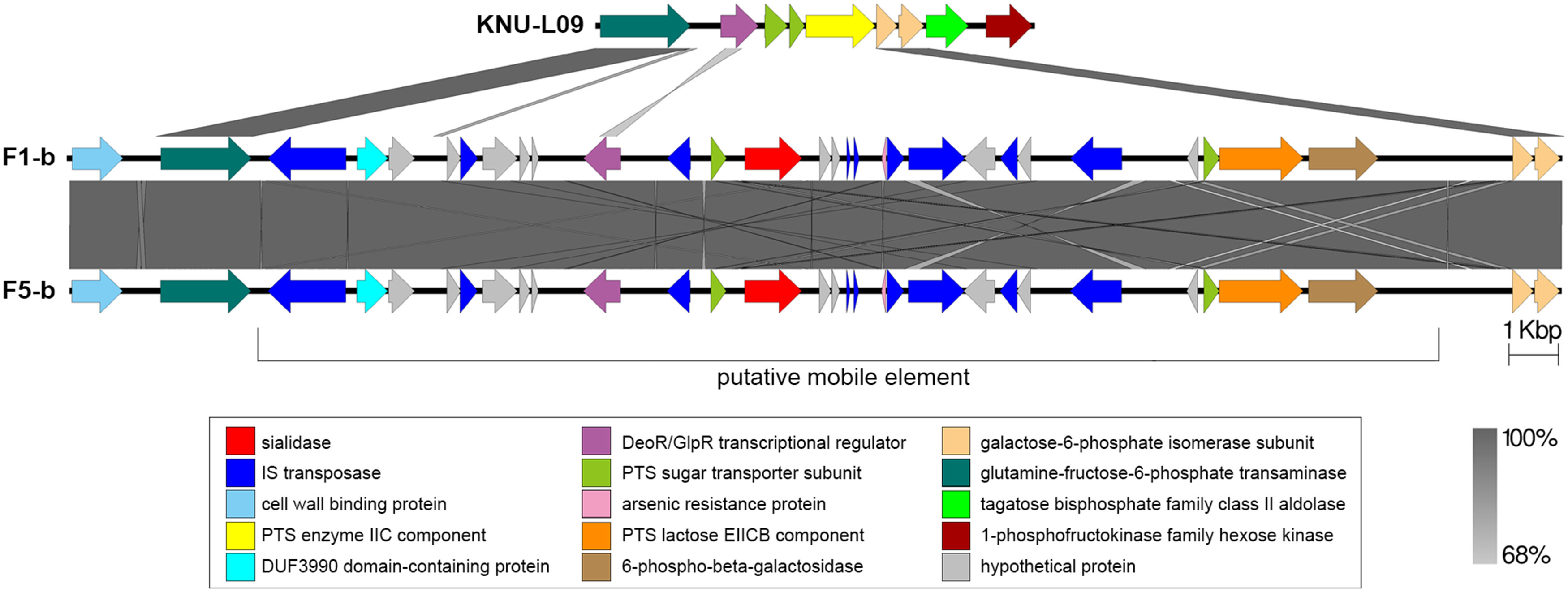
Genomic environment analysis of phosphotransferase system (PTS) lactose/transposon element which were extracted from whole-genome sequencing (WGS)-based analysis. EasyFig version 1.0 software was using to show the BLAST results. The IS transposases from left to right: IS*1182*, IS*66*, IS*200/605*, IS*91*, IS*256*, IS*3* and IS*30*. PTS sugar transporter subunit including PTS lactose/cellobiose transporter subunit IIA, PTS fructose transporter subunit IIB and PTS sugar transporter subunit IIA.

Two complete genomes were determined by PacBio RSII long-read sequencing chemistries to an average coverage of 163× for the F1-b and 137× for F5-b. The genome sequence of 2 *C. butyricum* isolates consisted of a single circular chromosome and 1 circular megaplasmid. The chromosome of *C. butyricum* F1-b consisted of 3,864,433 bp (3,864,393 bp for F5-b) with 3,472 (3,469 for F5-b) coding sequences. The average guanine-cytosine (GC) content was 28.88% for both genomes. The total size of the 1 plasmid from isolate F1-b was 882,416 bp (882,389 for F5-b), with an average GC content of 28.51% for both. For strain comparison, a reference strain *C. butyricum* KNU-L09 from NCBI was obtained (Supplementary Table S1 online). To identify potential virulence-encoding genes in the 2 strains of *C. butyricum*, these genomes were used to query those factors listed in the VFDB. No virulence genes were identified on the plasmids (Supplementary Fig. S1 online). The *nanH* gene was found in the chromosome of 2 isolates but was not present in the reference strain (Fig. 3). In particular, the *nanH* gene was located in a transposon: IS*1182*, IS*66*, IS*200/605*, IS*91*, IS*256*, IS*3*, and IS*30* were flanking it (Fig. 4). Virulence factors, including hemolysin A, hemolysin B, hemolysin C, and hemolysin III family protein identified previously by Cassir et al,^8^ enterotoxin identified by Kwok et al,^22^ were detected in both *C. butyricum* strains sequenced in this investigation along with the reference strain (Fig. 3 and Table 3).

**Table 3.** List of Virulence Genes in F1-b and F5-b

Gene Name	Locus Tag		Protein Name	Reference
	F1-b	F5-b		
*nanH*	EBL75_05455	EBQ27_05455	Sialidase	VFDB
*tlyA*	EBL75_11590	EBQ27_11595	Hemolysin A	Cassir et al^9^ 2015
*tlyB*	EBL75_04805	EBQ27_04805	Hemolysin B	Cassir et al^9^ 2015
*tlyC*	EBL75_20695	EBQ27_20700	Hemolysin C	Cassir et al^9^ 2015
…	EBL75_04095	EBQ27_04095	Hemolysin III family protein	Cassir et al^9^ 2015
*hylA*	EBL75_16000	EBQ27_16000	β-Hemolysin	Cassir et al^9^ 2015
…	EBL75_11210	EBQ27_11215	Enterotoxin	Kwok et al^23^ 2014

Note: “…” indicates that there was no gene name from Genbank file.

A phylogenetic tree based on the core genome alignment was constructed to analyze the evolutionary relationships between our 2 isolates and 16 *C. butyricum* strains from NCBI (Supplementary Fig. S2 online). The tree revealed that the 2 *C. butyricum* isolates were in the same clade, a finding consistent with the PFGE result.

## Discussion

Outbreak of NEC in neonates, especially in preterm neonates caused by *C. butyricum* is known, but globally, only a few have been reported. This outbreak of NEC occurred in a neonatal ward with evidence of association between the presence of toxic *C. butyricum* strains recovered in the stool samples taken from neonates and the occurrence of NEC. These results are in line with earlier reports.^13^ Those *C. butyricum* cultured from different environmental and stool samples during outbreak shared the same pulsotype, suggesting a common origin. To the best of our knowledge, this is the first report describing a NEC outbreak linked to the infection of *C. butyricum* in China.

Notably, some medical instruments, disposable items for neonate daily use, neonate food, and incubators that came into contact with the medical staff were all *C. butyricum* positive. All *C. butyricum* from these samples shared the same pulsotype with those isolates recovered from both swabs taken from the hands of the medical staff and NEC patient stools. Data matching led us to strengthen the involvement of *C. butyricum* in the occurrence of NEC independent of intrinsic factors. In particular, facilities and items in the ward that the medical staff touched were all contaminated with *C. butyricum*. These findings demonstrate that the medical staff themselves may have contributed to transmission of *C. butyricum* from the original source of contamination to these NEC cases. Additionally, breast milk, the inner wall of breast milk bags, as well as a box for holding breast milk bags were contaminated by *C. sporogenes* with indistinguishable PFGE profile. Thus, the contamination may have come from the breast milk due to mother’s poor hygiene habits or from breast milk collection equipment or materials. Although concurrent contamination of *C. butyricum* and *C. sporogenes* in some samples was detected, no literature-based evidence has linked NEC outbreaks with infections associated with *C. sporogenes*. On the other hand, no NEC cases were reported after the ward disinfection following the outbreak, and all samples collected were negative for *Clostridium*. It is tempting to speculate that the outbreak of NEC reported in the present study was associated with the nosocomial infection of *C. butyricum*.

NEC-like lesions have been found in animal models when *C. butyricum* cultures were administered orally.^9^^–^^12^ This may be due to the production of butyric acid by the bacterium, whose effect on the intestinal barrier is paradoxical. Butyrate at a low concentration (2 mM) promotes intestinal barrier function but significantly reduces it at a high concentration (8 mM).^9^ Thus, a working hypothesis to explain the events described herein involves the overproduction of short-chain fatty acids in the bowel, which in turn may play an important role in the pathogenesis of NEC.

Our WGS data indicated that sialidase, hemolysin, and enterotoxin virulence factors were located in the chromosome of both *C. butyricum*. The sialidase encoding gene was unique to these 2 isolates. Increasing evidence indicates that sialidase and sialic acid play significant roles in growth and colonization of the intestines by bacterial pathogens, such as *C. perfringens* and others.^23^^–^^29^ These factors can help the pathogen avoid the host immune system defense.^30^ The sialidase encoding gene *nanH* identified in this study has not been previously reported in *C. butyricum*.^31^^,^^32^ The translated protein product of this gene shares 75.853% identity with the *nanH* exo-alpha-sialidase from *C. perfringens* (accession no. ATD49144.1). Among those hemolysins detected in the 2 isolates, the pore-forming β-hemolysin has been described as the main virulence factor capable of inducing enterocyte necrotic lesions. Purified β-hemolysin affects the integrity of epithelial cell monolayers and induces colonic epithelial cell damage.^33^ Enterotoxin is likely responsible for the most common and important human gastrointestinal illnesses caused by *C. perfringens*.^34^ Many studies have reported that enterotoxigenic strains of *C. perfringens* have previously been related to enteritis in human and several animal species.^35^^–^^37^

Although the pathogenic mechanism of NEC elaborated by *C. butyricum* remains to be fully understood, based on these findings in our study, we hypothesize that the sialidase might promote the growth and colonizing of the intestines with *C. butyricum* and possibly protect it from the hosts immune system defense. The hemolysin and enterotoxin factors may then act to induce intestinal cell damage. Interestingly, we found the sialidase encoding gene located on a putative mobile genetic element, and this virulence gene might be transferred from other pathogens such as *C. perfringens*. This observation suggests that a similar mechanism may exist in the case of other clostridial species (ie, *C. perfringens*, *C. neonatale*).

Our study has some limitations. First, the stool samples were obtained from only 2 of 15 NEC patients because most patients (11 in total) had recovered fully and had been discharged from the hospital at the time of sampling and because feces samples produced by 2 patients were too small for analysis owing to fasting and water-free treatment. Secondly, only 2 representative strains of 15 *C. butyricum* cultured from samples linked to NEC cases were selected for WGS because all 15 strains shared the same PFGE profile. The remaining strains will be sequenced later to elucidate the genetic differences among them.

In conclusion, our findings show an association between NEC and *C. butyricum* contamination within the hospital. Monitoring of this bacterium in the neonatal ward and pediatric outpatient unit as well as good medical practices should be reinforced and strictly implemented. Moreover, a more extensive study to describe transmission routes, and the possible toxigenic mechanism of *C. butyricum* involved in the pathogenesis of NEC warrants further investigation.

## Supplementary material

For supplementary material accompanying this paper visit https://doi.org/10.1017/ice.2019.289. click here to view supplementary material
