# Supplementary material for: Epidemiological and genetic characterization of Clostridium butyricum cultured from neonatal cases of necrotizing enterocolitis in China
Source: Infect Control Hosp Epidemiol. 2020 Jun 16;41(8):900–7. doi: 10.1017/ice.2019.289 (PMC7511950; doi:10.1017/ice.2019.289)
Supplement: Supplementary file 1 [file S0899823X19002897sup.zip › S0899823X19002897sup001.docx]

**Supplementary** Fig. S1**.** Megaplasmid pangenome of *C.butyricum* F1-b and F1-5 compared with reference strain *C. butyricum* KNU-L09 (accession no. NZ_CP013252).

Beginning from the inside outward, the following features are represented: GC content (black); GC skew (green and purple); *C. butyricum* KNU-L09 plasmid (red); *C. butyricum* F1-b plasmid (green); *C. butyricum* F5-b plasmid (blue); transport genes (shown in green font), putative mobile element (shown in purple font), phage genes (shown in black font) and CRISPR-Cas (shown in light-blue font).

**Supplementary** Fig. S2. Phylogenetic tree of 2 *C. butyricum* isolates identified in this study with 16 *C. butyricum* strains from NCBI.
