# Supplementary material for: Epidemiological and genetic characterization of Clostridium butyricum cultured from neonatal cases of necrotizing enterocolitis in China
Source: Infect Control Hosp Epidemiol. 2020 Jun 16;41(8):900–7. doi: 10.1017/ice.2019.289 (PMC7511950; doi:10.1017/ice.2019.289)
Supplement: Supplementary file 1 [file S0899823X19002897sup.zip › S0899823X19002897sup004.docx]

Table S1 General comparative analysis of two *C. butyricum* isolates (F1-b, F5-b) and reference strain (*C. butyricum* KNU-L09)

| Features | *C. butyricum* F1-b | | *C. butyricum* F5-b | | *C. butyricum* KNU-L09 | |
| --- | --- | --- | --- | --- | --- | --- |
|  | Chromosome  (Accession CP033247) | Plasmid  (Accession CP033246) | Chromosome  (Accession CP033249) | Plasmid  (Accession CP033248) | Chromosome  (Accession NZ_CP013252.1) | Plasmid  (Accession NZ_CP013489.1) |
| No. of chromosome and plasmid | 1 | 1 | 1 | 1 | 1 | 0 |
| Genome size (bp) | 3,864,433 | 882,416 | 3,864,393 | 882,389 | 3,824,894 | 803,000 |
| G+C content (%) | 28.88 | 28.51 | 28.88 | 28.51 | 28.88 | 28.30 |
| No. of predicted coding sequences (CDS) | 3,472 | 844 | 3,469 | 837 | 3912 | 721 |
| No. of rRNA operons | 36 | 0 | 36 | 0 | 33 | 0 |
| No. of tRNA operons | 87 | 0 | 87 | 0 | 87 | 0 |
| No. of CRISPR regions | 0 | 0 | 0 | 0 | 0 | 1 |
