# Supplementary figures and images for: Epidemiological and genetic characterization of Clostridium butyricum cultured from neonatal cases of necrotizing enterocolitis in China
Source: Infect Control Hosp Epidemiol. 2020 Jun 16;41(8):900–7. doi: 10.1017/ice.2019.289 (PMC7511950; doi:10.1017/ice.2019.289)

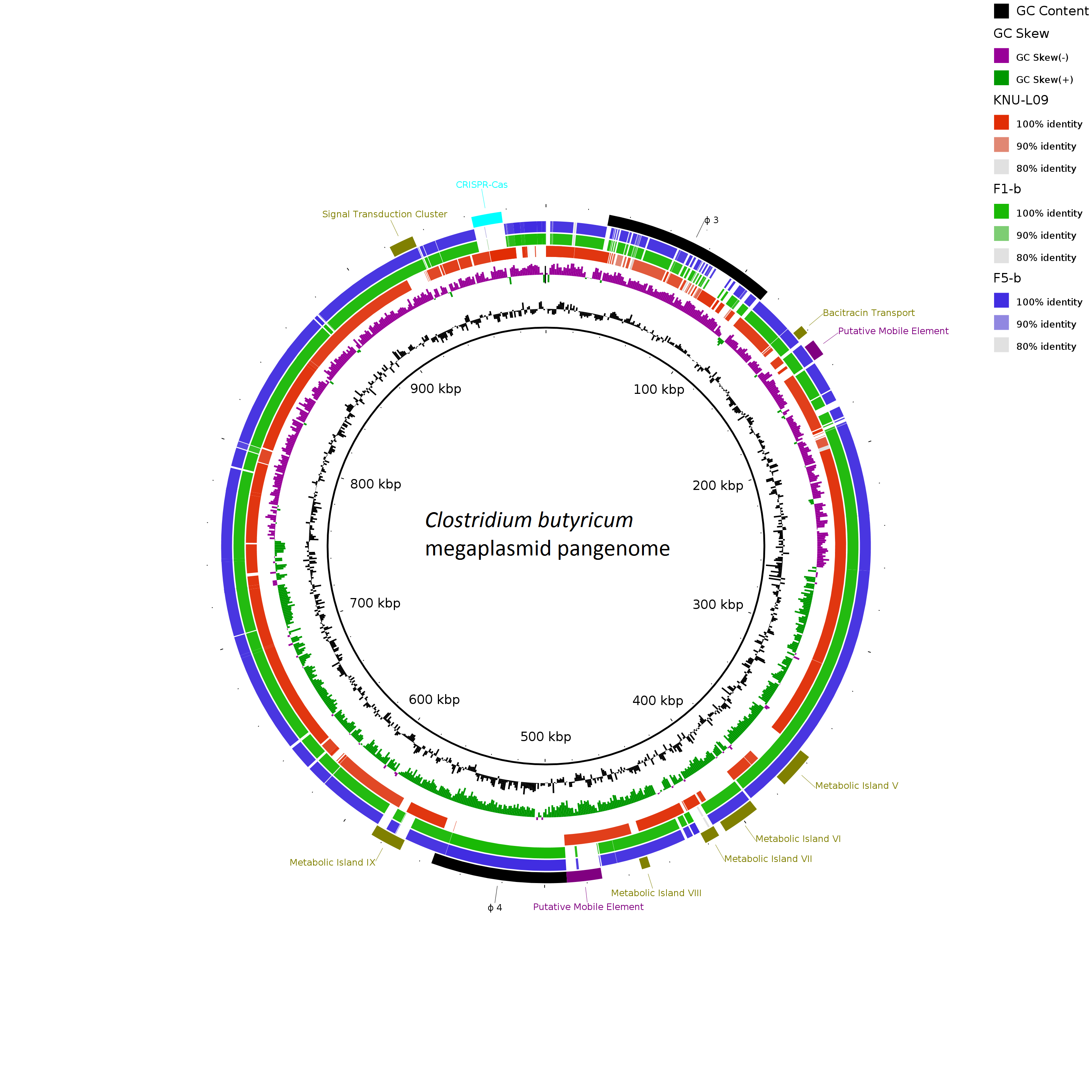

Supplement: Supplementary file 1 [file S0899823X19002897sup.zip › S0899823X19002897sup002.png]

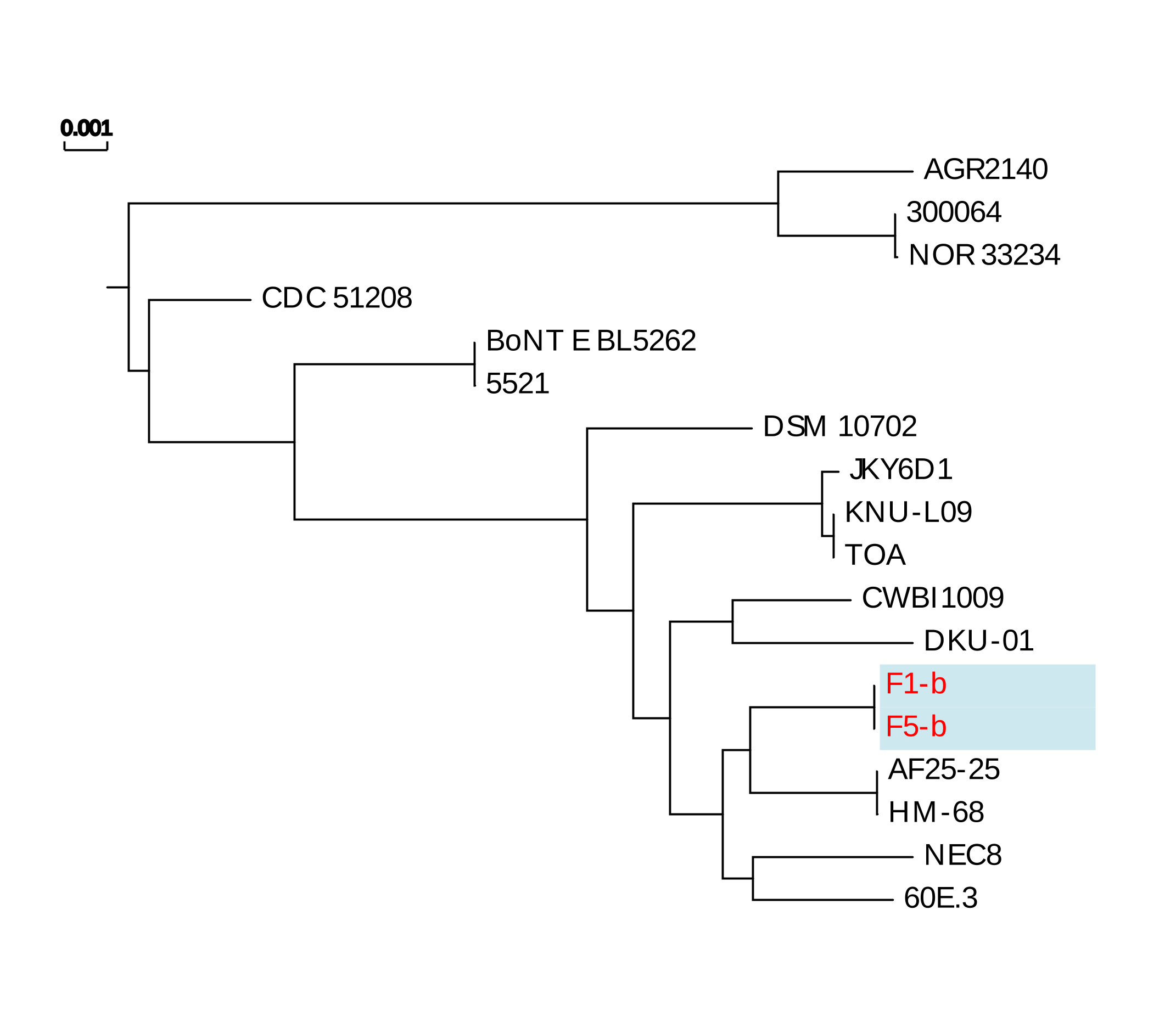

Supplement: Supplementary file 1 [file S0899823X19002897sup.zip › S0899823X19002897sup003.tiff]
